# Physical activity ameliorates cardiovascular health in elderly subjects: the functional role of the β adrenergic system

**DOI:** 10.3389/fphys.2013.00209

**Published:** 2013-08-12

**Authors:** Gaetano Santulli, Michele Ciccarelli, Bruno Trimarco, Guido Iaccarino

**Affiliations:** ^1^Department of Translational Medical Sciences, “Federico II” UniversityNaples, Italy; ^2^Department of Advanced Biomedical Sciences, “Federico II” UniversityNaples, Italy; ^3^College of Physicians and Surgeons, New York Presbyterian Hospital, Columbia University in the City of New YorkManhattan, New York, NY, USA; ^4^Department of Medicine and Surgery, University of SalernoSalerno, Italy; ^5^Multimedica Research HospitalMilan, Italy

**Keywords:** aging, beta adrenergic system, elderly, physical exercise, heart failure, hypertension, atrial fibrillation, elderly population

## Abstract

Aging is a complex process characterized by a gradual decline in organ functional reserves, which eventually reduces the ability to maintain homeostasis. An exquisite feature of elderly subjects, which constitute a growing proportion of the world population, is the high prevalence of cardiovascular disorders, which negatively affect both the quality of life and the life expectancy. It is widely acknowledged that physical activity represents one of the foremost interventions capable in reducing the health burden of cardiovascular disease. Interestingly, the benefits of moderate-intensity physical activity have been established both in young and elderly subjects. Herein we provide a systematic and updated appraisal of the literature exploring the pathophysiological mechanisms evoked by physical activity in the elderly, focusing on the functional role of the β adrenergic system.

## Introduction

Aging is a multifaceted process characterized by a gradual decline in organ functional reserves (Santulli and Iaccarino, 2013; Trindade et al., 2013). Numerous theories of aging have been proposed hitherto and all of them somehow relate the lifespan of each single species to mechanisms essential for the maintenance of several biological activities (Raj et al., 2012). The free radical theory relates to the failure of defenses against reactive oxygen species (Davis and Williams, 2012; Joiner et al., 2012); the somatic mutation theory relates to an impaired DNA repair (Agronin, 2013); the autoimmune theory proposes that the immune system fails to distinguish self from non-self antigens (Menconi et al., 2008); others relate aging to the loss of epigenetic controls, including but not limited to DNA methylation, or to detrimental effects of toxic chemicals (Sahin et al., 2011; Akbarian et al., 2013; Santulli and Totary-Jain, 2013).

Although the process seems to be continuous and irreversible, aging itself does not mean disease. Indeed, aging is not a pathological condition but an entirely natural phenomenon. Age-linked modifications, however, whose mechanisms have to be looked at the cellular and molecular level, indubitably pave the way for disease (Marks, 2008).

## Aging of cardiovascular system

Aging is associated with evident changes in the cardiovascular system that reflect alterations of biochemical adaptive mechanisms (Santulli and Iaccarino, 2013). Normal aging, even in the absence of co-morbidities, results in cardiovascular stiffening (Lakatta, 2003; Santulli, 2012b).

### The heart

Significant alterations of measures of ventricular filling and relaxation have been described with aging, including a reversal of the early and late mitral inflow velocities (E/A ratio), a prolongation of isovolumetric relaxation time, a modification of the dynamic longitudinal wall relaxation, and diastolic suction (propagation velocity of early mitral inflow). All these differences have been reported across a wide range of physiological filling pressures (Prasad et al., 2007).

The incidence of left ventricular hypertrophy (LVH), heart failure (HF), and atrial fibrillation (AF) increases dramatically with age (Santulli, 2013). In particular, the prevalence of LVH also increases with rising blood pressure and body mass index (Lanni et al., 2007; Sorriento et al., 2010; de Simone et al., 2013). Cross-sectional studies of subjects without hypertension indicate that left ventricular wall thickness increases progressively with age. Moreover, in older hospitalized patients without clinically apparent cardiovascular disease, in whom overall cardiac mass was not increased, cardiomyocyte enlargement was observed at autopsy (Olivetti et al., 1995).

The elderly appear to be particularly predisposed to the development of HF. Such a diagnosis is indeed the leading cause of hospitalizations in people >65 years of age (Rosca and Hoppel, 2010; Santulli, 2013). In seniors, the underlying substrate for HF, particularly with preserved ejection fraction (occurring in about one-third to one-half of older patients with HF), may in part be the age-associated modification in ventricular compliance and relaxation (Marin-Garcia and Goldenthal, 2008; Oghlakian et al., 2011). Albeit such mechanical changes may not be enough to explain the cause of HF with preserved systolic function by themselves, superimposed conditions including diabetes, coronary disease, or hypertension may tip the scale toward increased filling pressures and pulmonary congestion (Prasad et al., 2007; Ahuja et al., 2013; Hohendanner et al., 2013; Kohlhaas and Maack, 2013). Regular lifelong physical activity preserves cardiac compliance (in the absence of co-morbid conditions) and thereby decreases left ventricular end-diastolic pressure (Prasad et al., 2007).

Lastly, AF is detected in approximately 3–4% of healthy volunteers over age 60 years without clinical coronary artery disease. Such a rate is 10-fold higher than in the general adult population (Santulli et al., 2012b). Overall prevalence of AF has been quantified to be 17.8% in people aged 85 years and above (Santulli, 2013). The lifetime risk to develop AF at the age of 55 years is 23.8% in men and 22.2% in women (Boriani et al., 2006; Santulli, 2013). Notably, the development of a rapid irregular pattern of electrical activity, including AF, may have detrimental consequences for hearts that are relatively stiff and relax slowly (D'Ascia et al., 2011; Santulli et al., 2012c; Du et al., 2013; Santulli, 2013).

### The vessels

At the vascular level, cross-sectional studies in humans have shown that wall thickening and dilatation are prominent structural changes that occur within large elastic arteries during aging. The carotid intimal-media thickness increases 2- to 3-fold between 20 and 90 years of age (Lakatta, 2003). The aortic wall thickening that occurs with aging consists mainly of intimal thickening (Virmani et al., 1991). Age-associated increase in intimal thickening is accompanied by both luminal dilatation and a reduction in distensibility or compliance, resulting in an increase in vessel stiffness. Pulse wave velocity (PWV), a relatively convenient, non-invasive index of stiffening (Safar et al., 2005), increases with age. Such increase is determined in part by the intrinsic stress/strain relationship (stiffness) of the vascular wall and by the mean arterial pressure. Augmented PWV has traditionally been linked to structural alterations in the media, including increased collagen, reduced elastin content, elastin fractures, and calcification. Prominent age-associated increases in PWV have been demonstrated in populations with little or no atherosclerosis, thus indicating that stiffening can occur independently of atherosclerosis (Boutouyrie et al., 2002). However, more recent data emerging from epidemiological studies indicate that increased large vessel stiffening also occurs in the context of atherosclerosis and diabetes (Milan et al., 2011; Kollias et al., 2012). A potential link might be that stiffness is governed not only by the structural changes within the matrix, as noted above, but also by endothelial regulation of smooth muscle tone and of other aspects of vascular wall structure/function (Costanzo et al., 2010). Of interest, abnormalities of the endothelium have been identified to occur early on in the pathophysiology of atherosclerosis, diabetes, and hypertension (Iaccarino et al., 2004; Santulli et al., 2012a,e).

## Physical activity and cardiovascular system

Physical training is associated with improvements in blood pressure regulation, lipid profile, abdominal fat reduction, insulin sensitivity, and hemodynamic, inflammatory and psychosocial parameters (Piepoli et al., 2004; Woodman et al., 2005; Korantzopoulos and Goudevenos, 2007; Niederseer et al., 2007; Werner et al., 2009; Berry et al., 2012). In addition, engaging in physical activity of any intensity (including low-intensity ones) likely positively impacts insulin action and blood glucose control acutely (Colberg, 2012; Santulli et al., 2012e). Aerobic exercise training may significantly lower blood pressure in older hypertensive individuals, improving endothelial function and vascular tone (Hagberg et al., 1989; Dengel et al., 2006). In particular, swimming exercise has been shown to elicit hypotensive effects and improvements in vascular function in previously sedentary older adults (Nualnim et al., 2012). A recent meta-analysis explored the effects of different kinds of physical exercise on blood pressure in adult subjects (Cornelissen and Smart, 2013). Combined training was shown to lower only diastolic blood pressure, whereas endurance, dynamic resistance, and isometric resistance training lowered both systolic and diastolic blood pressure. Isometric resistance training appeared to have the potential for the largest reductions in systolic blood pressure.

### Effects of exercise on skeletal muscle

Life-long endurance exercise training has been shown to prevent age-associated declines of exercise capacity and cardiac compliance in healthy subjects (Arbab-Zadeh et al., 2004). Moreover, several months to a year of exercise training can increase exercise capacity in healthy subjects (Fujimoto et al., 2010) and HF patients (Sullivan et al., 1988). The improved exercise capacity in HF appears to be related to improvements in peripheral arterial function and skeletal muscle metabolism (Beere et al., 1999). A single bout of strenuous exercise in a previously sedentary subject unleashes a broad array of cellular and molecular processes, which serve to quickly prepare for the next episode of physical exertion. The opposite can also occur quickly, as evidenced by the marked muscle atrophy and decline in exercise performance following forced bed rest in older individuals, which likely mimics the condition resulting from the frequent hospitalizations experienced by older HF patients (Kitzman and Haykowsky, 2012).

### Workload and cardiac performance

Coats and colleagues proved in a milestone study that home-based physical training programs are feasible even in severe chronic HF and have a beneficial effect on exercise tolerance, peak oxygen consumption, and symptoms (Coats et al., 1990). Indeed, heart rates at submaximal workloads and rate-pressure products are significantly reduced by training, and a significant improvement in patient-rated symptom scores has been also reported. Such findings have been confirmed by other investigators (Hambrecht et al., 2000; Kemps et al., 2010), demonstrating that exercise training in patients with stable HF improves the work capacity by enhancing endothelial function and skeletal muscle aerobic metabolism. In addition, physical exercise is associated with reduction of peripheral resistance and results in small but significant improvements in stroke volume and reduction in cardiomegaly. In a very well-designed study Belardinelli and colleagues recently demonstrated that moderate supervised training, at 60% of peak oxygen consumption (VO_2_), performed twice weekly for 10 years confers a sustained improvement in quality of life compared with non-trained patients (Belardinelli et al., 2012). Such sustained improvements are associated with reduction in major cardiovascular events, including hospitalizations for HF and cardiac mortality (Belardinelli et al., 2012; Santulli, 2013) and a sustained improvement in quality of life (Khazanie and Granger, 2013). Thus, the commonly held belief that rest is the mainstay of treatment of chronic HF should no longer be accepted.

### Exercise and cardiac structure

Diastolic dysfunction has been strongly related to decreased exercise capacity in a large population referred for exercise echocardiography and not limited by ischemia, (Grewal et al., 2009). Increased resting and post-exercise left ventricular filling pressures have been also associated with a reduction in exercise capacity. Of note, unlike many other factors that are an inevitable consequence of aging, diastolic dysfunction may be a preventable factor in the development of exercise intolerance. However, it is not completely clear whether alterations in several markers of diastolic function with senescence are a specific manifestation of the aging process or reflect a secondary cardiac adaptation to a more sedentary lifestyle. This issue has been elegantly addressed by Prasad and colleagues, who demonstrated that, in contrast to chamber compliance, age-dependent modifications of ventricular relaxation are only minimally influenced by lifelong endurance training (Prasad et al., 2007). Hence, changes in ventricular compliance with senescence are strongly influenced by physical activity, whereas changes in ventricular relaxation appear to be more likely specific to cardiac senescence and may result from alterations in cardiac regulatory proteins that occur with aging (Loffredo et al., 2013).

### Physical activity and atrial fibrillation

There are currently controversial results concerning the effect of physical training on AF. In young and middle-aged adults, high-intensity endurance training is associated with higher risk of lone AF (Molina et al., 2008; Mont et al., 2008). On the other hand, a recent report indicated that greater habitual light-to-moderate physical activities are associated with significantly lower risk of new-onset AF in older adults (Mozaffarian et al., 2008). Several factors may explain such apparently divergent findings. AF is a common clinical manifestation of remarkably heterogeneous cardiac and non-cardiac conditions, including coronary artery disease, valvular disease, hypertension, sleep apnea, alcohol use, pericarditis, hyperthyroidism, and genetic predisposition (D'Ascia et al., 2011; Santulli and D'Ascia, 2012). Lone AF should exclude subjects with hypertension, clinical or structural cardiopulmonary disease, or age >60 years; hence, lone AF explicitly does not exist in older adults (Fuster et al., 2006; Santulli, 2011). Pathophysiology of lone AF, which represents ≤10% of AF cases in the population, may be indeed very different from the much more common AF seen with structural heart disease, hypertension, other disorders, or aging. Thus, physical training could increase incidence of lone AF in young and middle-aged adults but may also attenuate numerous other AF risk factors reducing overall incidence of AF, particularly later in life when risk rises so steeply (Santulli et al., 2012d). Furthermore, activity intensity might modify effects on AF, depending on the balance between acute triggering versus reduction of chronic vulnerability to AF. Then, since nearly 1 in 5 subjects aged ≥65 years are supposed to develop AF during the next 10 years (Mozaffarian et al., 2008), habitual light to moderate physical activity might be an exceptional prescription to help lower such a risk.

## Aging, physical exercise, and beta adrenergic system: the molecular evidence

### Cardiovascular β adrenergic system

Several experimental findings indicate an age-associated decrease in catecholamine-responsiveness in the elderly. In particular, an age-associated decrease in β adrenergic receptor (βAR) sensitivity and density has been shown in the cardiac muscle and has been mainly attributed to down-regulation and impaired coupling of βAR to adenylate cyclase (Lakatta, 2003). The age-linked decline in cardiac βAR response, which is consistent across species, seems to be primarily due to a down-regulation of β_1_ARs, as reported in aged explanted human hearts (White et al., 1994). Further, a reduction in the sensitivity of βARs, measured by isoproterenol-induced changes in the catecholamine stimulated adenylate cyclase activity in the myocardium (O'Connor et al., 1981) and in pulse rate and blood pressure (Vestal et al., 1979), had been reported.

Young individuals are more responsive than elderly subjects to isoproterenol-induced increases in blood flow in the brachial artery (van Brummelen et al., 1981). Such features are similar to what seen in patients with HF. Hence, most of the modifications that occur in the sympathetic nervous system with aging (hyposensitivity to adrenergic stress, increased circulating catecholamines and decreased βAR responsiveness) are also common in HF patients (Santulli, 2012b).

### Aging and βAR signaling abnormalities

The age-linked decline in adrenergic responsiveness impairs also vasodilatation, increasing thereby total peripheral resistances (Santulli and Iaccarino, 2013). A generalized impairment of βAR-mediated vasorelaxation has been indeed shown both in human hypertensive patients (Izzo et al., 2008) and in animal models of hypertension (Borkowski et al., 1992; Iaccarino et al., 2004; Santulli et al., 2009). The age-associated decrease in βAR-mediated relaxation has been proposed to be due to decreased receptor density, less efficient coupling to adenylate cyclase, impaired generation of cyclic AMP, or attenuated activation of downstream components (Santulli et al., 2013). However, there is not a single factor that can entirely explain the age-related deterioration of βAR function (Santulli et al., 2011a; Lampri and Elli, 2013). The primary trigger of such homeostatic imbalance seems to be associated with an age-related alteration in the ability of βAR to respond to agonists at the cellular level. βAR affinity for the ligand is dependent upon its phosphorylation, which in turn is in the domain of G protein-coupled receptor kinases (GRKs) and GRK2 in particular (Santulli et al., 2011b; Fusco et al., 2012). Intriguingly, both GRK2 expression and activity increase in vascular tissue with aging (Santulli et al., 2013). Furthermore, the transgenic overexpression of GRK2 in the vasculature leads to impaired βAR signaling and vasodilatative response, causing a hypertensive phenotype in rodents Such a point of view has been supported in humans by the observation that GRK2 expression correlates with blood pressure as well as impaired βAR-mediated adenylate cyclase activity (Sorriento et al., 2012; Santulli et al., 2013). The deterioration in βAR function and subsequent cAMP generation (Davinelli et al., 2012) is a common factor underlying hypertension, atherosclerosis, vascular insufficiency, and orthostatic hypotension, all conditions associated to important morbidity and mortality (Santulli, 2012a; Vu et al., 2012).

### Physical exercise and βAR

The βAR system is activated in lymphocytes during prolonged aerobic physical exercise both in healthy subjects and in HF patients (Maki, 1989; Mancini et al., 1989). The number of lymphocyte βAR increases after dynamic exercise by a β_2_AR mechanism (Maisel et al., 1990). Such increase can indeed be mimicked by acute administration of exogenous βAR agonists such as isoproterenol and epinephrine but not by norepinephrine (Hazeki, 1973; Deblasi et al., 1986). Moreover, it can be blocked by non-selective βAR antagonists such as propranolol and the β_2_AR-selective ICI 118,551 but not by the β_1_AR-selective bisoprolol (Van Tits et al., 1990).

The effects of exercise on cardiovascular catecholamine responsiveness have been extensively studied, pointing out the functional role of βAR. An increased responsiveness to isoproterenol has been demonstrated in the myocardium of trained rodents, when compared with sedentary controls (Takeda et al., 1985; Libonati and MacDonnell, 2011). Such responses have been shown to be independent of training-induced alterations in cardiac hypertrophy or hypertrophic marker expression. An increased sensitivity of β_2_AR has been indicated as a mechanism underpinning the increased vasodilator response to isoproterenol after exercise (Gaballa et al., 2000; Santulli and Iaccarino, 2013). Several studies have also shown the importance of the βAR system in the relaxant response of coronary arteries during exercise. Indeed, the β_2_AR receptor selective antagonist ICI 118,551 is able to significantly decrease coronary blood flow velocity and increase late diastolic coronary resistance during a running session (DiCarlo et al., 1988; Traverse et al., 1995).

Other recent studies have shown in animal models that physical exercise ameliorates sensitivity of βAR when the vasodilator response mediated by such receptors has been previously reduced by the aging process. In particular, a 6-week training program of 5 days/week swimming exercise improved vasodilator response to the non-selective βAR agonist isoproterenol, in coronary arteries, compared with the sedentary group (Santulli and Iaccarino, 2013). Similarly, a 10- to 12-week treadmill program of 5 days/week running exercise, with 60-min sessions, improved vasodilator response to isoproterenol in gastrocnemius muscle vessels from old rodents, but not in young animals (Donato et al., 2007).

Collectively, the aforementioned studies show the beneficial effects of physical training on vascular sensitivity in the aging process. However, βAR responsiveness to exercise is not homogeneous, depending on several factors, including the region of vascular bed to be studied. Indeed, regions of different diameters in the same artery might respond differently to physical exercise. Another noteworthy variable is the type of artery studied: resistance and conductance vessels show indeed different responses.

## Summary

Aging has become one of the most critical issues for industrialized nations, because population average age, and thus the incidence of age-associated disorders, has markedly risen to create a major burden as patients draw heavily on the need for continuing medical treatment and hospital and other community services. In the present review, we examined the relationship between physical training and aging, focusing on the functional role of βARs. The amelioration of βAR responsiveness, obtained through means of regular physical training, contributes to the clinical improvement in cardiovascular health reported in elderly subjects.

### Conflict of interest statement

The authors declare that the research was conducted in the absence of any commercial or financial relationships that could be construed as a potential conflict of interest.
